# The Relationship Between Decreased CD-8 T-Cells and Mortality in Patients with COVID-19 Pneumonia in the Intensive Care Unit, A Retrospective Study

**DOI:** 10.4274/TJAR.2022.22959

**Published:** 2023-06-16

**Authors:** Zeynep Tuğçe Sarıkaya, Bülent Güçyetmez, Ayşe Sesin Kocagöz, Lütfi Telci, İbrahim Özkan Akıncı, COVID-19 Study Group

**Affiliations:** 1 Department of Anaesthesiology and Reanimation, Acıbadem Mehmet Ali Aydınlar University Faculty of Medicine, İstanbul, Turkey; 2 General Intensive Care Unit, Acıbadem Altunizade Hospital, İstanbul, Turkey; 3 General Intensive Care Unit, Acıbadem International Hospital, İstanbul, Turkey; 4 Department of Infectious Diseases and Clinical Microbiology, Acıbadem Mehmet Ali Aydınlar University Faculty of Medicine, İstanbul, Turkey; 5 General Intensive Care Unit, Acıbadem Maslak Hospital, İstanbul, Turkey; 6 General Intensive Care Unit, Acıbadem Taksim Hospital, İstanbul, Turkey; 7 Department of Infectious Diseases and Clinical Microbiology, Acıbadem Altunizade Hospital, İstanbul, Turkey; 8 Department of Infectious Diseases and Clinical Microbiology, Acıbadem International Hospital, İstanbul, Turkey

**Keywords:** CD-8, COVID-19, critical care, mortality, pneumonia, T-cell

## Abstract

**Objective:**

CD-8 T-cells are responsible for the clearance of virally infected cells. In patients with Coronavirus disease-2019 (COVID-19) pneumonia, there are quantitative reductions and functional impairments in T-cells. Low CD-8 T-cell levels cause worse clinical situations. In this study, the relationship between decreased CD-8 T-cells and mortality in patients with COVID-19 pneumonia in the intensive care unit (ICU) was investigated.

**Methods:**

In this multicenter retrospective study, 277 patients were analyzed. Demographic data, ICU admission scores, blood gas levels, laboratory samples, and outcomes were recorded. Statistical Package for the Social Sciences version 28 was used for statistical analysis.

**Results:**

Two hundred forty of 277 patients were included in the study. The mortality rate was 43.3%. In non-survivors, median values of age, Charlson comorbidity index, Acute Physiology and Chronic Health Evaluation II (APACHE-II), procalcitonin, leukocyte count, neutrophil count, neutrophil-lymphocyte count ratio, and duration of invasive mechanical ventilation were significantly higher, whereas median values of PaO_2_-FiO_2_ ratio, lymphocyte count, CD-4, and CD-8 T-cells were significantly lower than those in survivors. In the multivariate Cox regression model, the risk of mortality increased 1.04-fold (1.02-1.06) and 1.05-fold (1.01-10.8) by every one unit increase in age and APACHE-II, respectively, whereas it decreased 0.71-fold (0.58-0.87) by every hundred increase in CD-8 T-cells *P* < 0.001, *P*=0.007 and *P*=0.001 respectively.

**Conclusion:**

According to our findings, age, APACHE-II, and CD-8 T-cell levels seem to be independent risk factors for mortality in patients with COVID-19 pneumonia in the ICU.

**Main Points**

• SARS-CoV-2 infection can cause pneumonia, which can lead to life-threatening acute respiratory failure (ARF) and acute respiratory distress syndrome (ARDS).

• In patients with Coronavirus disease-2019 (COVID-19) pneumonia, T-cells may decrease or their function may be impaired.

• According to our findings, age, Acute Physiology and Chronic Health Evaluation-II (APACHE-II) and CD-8 T-cell level seem to be independent risk factors for mortality in patients with COVID-19 pneumonia in the intensive care unit (ICU).

• It should be kept in mind monitoring the CD-8 T-cell level could be a marker for subsequent infections and treatment modalities.

## Introduction

CD-8 T-cells play a crucial role in viral infection control in adaptive immunity by destroying virus-infected cells and generating effector cytokines.^1^ Firstly, they are activated via their T-cell receptor by dendritic cells.^2^ After this activation, naive T-cells are differentiated by [interleukin (IL)-12], IL-2 and type-1 interferon (INF) and they have effector functions as cytotoxic granules (perforin and granzymes), tumor necrosis factor-_µ_, (TNF_µ_) (T-cell proliferation and target cell necrosis) and INF (IL-12 production, phagocytosis and increase in MHC-I and MHC-II).^3,4^ Activated CD-8 T-cells are able to kill and eradicate infected cells and provide protection against infection.^2^ During the Coronavirus disease-2019 (COVID-19) pandemic, it has been shown that there were quantitative reductions and functional impairments in T-cells in patients with COVID-19 pneumonia.^3^ Moreover, it was demonstrated that hospitalized COVID-19 patients with low CD-8 T-cell levels had worsened clinical status and increased mechanical ventilation (MV) and intensive care unit (ICU) needs.^5^ However, there are no specific data about the relationship between CD-8 T-cell levels and outcomes in patients with COVID-19 in the ICU. Therefore, we aimed to investigate the relationship between CD-8 T-cell levels and mortality in patients with COVID-19 pneumonia in the ICU.

## Methods

### Study Population

After approvals from the Scientific Committee of the Turkish Health Ministry (2020-08-21T18-17-45) and the Local Ethics Committee (ATADEK-2020/19), the study was retrospectively designed in 4 general tertiary ICUs between March 2020 and December 2020. Acıbadem Mehmet Ali Aydınlar University Medical Research Evaluation Board (ATADEK) (approval no: 2020-19/8, date: 03.09.2020). All data were fully anonymized without restriction after ethics committee approval, and the ethics committee waived the requirement for informed consent.

Two hundred seventy-seven patients with COVID-19 pneumonia were retrospectively evaluated. Patients whose T-lymphocyte subtypes were not studied, who were <18 and more than 90 years old or transferred to another center, who had human immunodeficiency virus (+) and who were administered immunosuppressive drugs were excluded from the study.

The patients were admitted to the ICU due to acute hypoxemic respiratory failure due to COVID-19 pneumonia resistant to conventional oxygen therapy and other accompanying organ failures. All patients were treated with antiviral drugs (favipiravir, hydroxychloroquine, azithromycin) and anticoagulant prophylaxis in accordance with the Turkish Health Minister’s Algorithm for COVID-19. Since early pandemic patient data were collected, it was revealed that there were no patients receiving steroids.

### Database

All data were collected from the Acıbadem Health Group Database. At the ICU admission, demographic data (age, body mass index, sex), charlson comorbidity index (CCI) and Acute Physiology and Chronic Health Evaluation II (APACHE-II) scores, P_a_O_2_-FiO_2_ ratio, C-reactive protein (mg dL^-1^), and procalcitonin (µg L^-1^) levels were collected; in the first week, maximum (max)-leukocyte count (leu_C_) (x10^3^ µL^-1^), max-neutrophil count (neu_C_) (x10^3^ µL^-1^), max-neutrophil-lymphocyte count ratio (NLCR), max-ferritin (ng mL^-1^), max-D-dimer (mg L^-1^), max-interleukin-6 (IL-6) (pg mL^-1^) levels and minimum (min)-lymphocyte count (lym_C_) (x10^3^ µL^-1^), min-CD-4 (µL^-1^) and min-CD-8 (µL^-1^) T-cells levels were collected. During the ICU period, culture results, the usage of IL-6 (tocilizumab, Actemra^®^, Switzerland) blocker, duration of invasive mechanical ventilation (IMV) (days), length of ICU stay (days), and mortality were also recorded. CD-4 and CD-8 T-cell levels were acquired using BD FACSCanto^TM^ (Erembodegem, Belgium) device, which employs a flow cytometry system.

### Statistical Analysis

Statistical Package for the Social Sciences version 28 was used for statistical analysis. The Kolmogorov-Smirnov test was used to detect normal distributions. Descriptive statistics were given as mean ± standard deviation, median (quartiles) and percentages. Student’s *t*-test, Mann-Whitney U, and chi-square tests were used for group comparisons. For the risk of mortality, age, CCI, APACHE-II, P_a_O_2_-FiO_2_ ratio, procalcitonin, max-leu_C_, max-neu_C_, max-NLCR, min-lym_C_, min-CD-4, and min-CD-8 T-cells were added to the univariate Cox regression analysis. Significant variables in the univariate analysis were added to the multivariate Cox regression model and the forward stepwise (likelihood ratio) method was used in the model. ROC analysis was used to detect the cut-off value for CD-8 T-cells for mortality. Two groups were determined in accordance with the cut-off value of CD-8 T-cells. In both patients and the low CD-8 T-cell patient group, Kaplan-Meier analysis was used to show the effect of the use of IL blockers on survival. Relative risks were calculated to identify the impact of the administration of IL-blockers on secondary infections in the low CD-8 T-cell subgroup. A *P* value < 0.05 was considered statistically significant.

## Results

Two hundred forty out of 277 patients were included in the study. In these patients, the mortality rate was 43.3% (Table 1). In nonsurvivors, age, CCI, APACHE-II, procalcitonin, max-leukocyte count, max-neutrophil count, max-NLCR and duration of IMV were significantly higher, whereas median values of PaO_2_-FiO_2_ ratio, min-lymphocyte count, min-CD-4 and min-CD-8 T-cells were significantly lower than survivors (Table 2). For mortality, the cut-off value of min-CD-8 T-cell level was detected as ≤115 (µL^-1^) (*P* < 0.001). In patients with CD-8 T-cell levels ≤115, age, CCI, APACHE-II, NLCR, CD-4/CD-8 ratio, mortality rate, min-lymphocyte counts, min-CD-4, and min-CD-8 T-cell levels were significantly different from those in patients with CD-8 T-cell >115 (Table 3).

**Figure 1:**
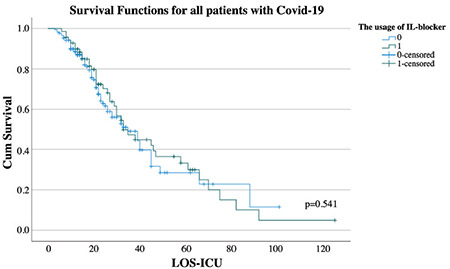
Survival functions for all patients with COVID-19.

**Figure 2:**
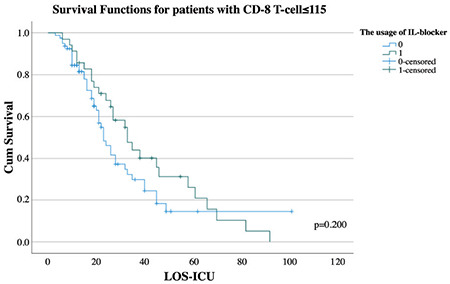
Survival functions for patients with CD-8 T-cell ≤115.

In the univariate cox regression model, the risk of mortality was significantly increased 1.05-fold (1.03-1.06), 1.01-fold (1.00-1.01), 1.05-fold (1.02-1.08) and 1.20-fold (1.11-1.30) by one unit increase in the age, NLCR, APACHE-II and CCI respectively whereas it was significantly decreased 0.79-fold (0.66-0.95), 0.84-fold (0.75-0.95) and 0.85-fold (0.77-0.93) by every hundred increase in CD-8 T-cell, CD-4 T-cell and min-lym_C_ respectively (*P* < 0.001, *P* < 0.001, *P* < 0.001, *P* < 0.001, *P*=0.011, *P*=0.007 and *P* < 0.001 respectively) (Table 4). In the multivariate cox regression model, the risk of mortality was increased 1.04-fold (1.02-1.06) and 1.05-fold (1.01-1.08) by one unit increase in age and APACHE-II respectively whereas it was decreased 0.71-fold (0.58-0.87) by every hundred increase in CD-8 T-cell (*P* < 0.001, *P*=0.007 and *P*=0.001 respectively) (Table 4). In Kaplan-Meier survival analysis, there was no effect of the usage of IL blockers on survival in either all patients or the patients with CD-8 T-cell ≤115 (*P*=0.541 and *P*=0.200) (Figures 1 and 2). Furthermore, the relative risk of having more than one group of microorganisms throughout the ICU stay was 1.4-fold (1.1-1.8) greater in patients with CD-8 T-cell levels ≤115 and IL blockers (+) than in patients with CD-8 T-cell levels >115 and IL blockers (-) (*P*=0.018).

## Discussion

In this study, our findings strongly suggest that nonsurvivors are older, have more comorbidities, have the worst clinical status at ICU admission, have severe alveolar-capillary damage, and have poor immunity. However, only age, APACHE-II, and CD-8 T-cell level are independent risk factors for mortality in patients with COVID-19 pneumonia in the ICU.

Lymphopenia and its relationship with the worst outcome are known in patients with COVID-19.^6,7^ There are two theories for this lymphopenia: the directly lethal effect of the virus on lymphocytes and the exhaustion of lymphocytes.^5,8^ If there is a direct lethal effect, it can be expected that lymphocytes and their subgroups will be affected in the same way. Peng et al.^9^ emphasized the importance of lymphocyte subgroup responses for recovery in patients with COVID-19. Iannetta et al.^10^ also showed the relationship between T-cell subgroup levels and mortality in COVID-19 patients. However, Urra et al.^5^ demonstrated that CD-8 T-cells were much more influenced than CD-4 T-cells. Interestingly, we observed lymphopenia (<1.3x10^3^ µL^-1^) in almost all patients (96.7%), whereas patients with low CD-8 (<200 µL^-1^) and CD-4 T-cell levels (<300 µL^-1^) accounted for only 75.8% and 70.4% of all patients, respectively. The reason for the difference in lymphocyte subgroups may be due to the more lethal effect on CD-8 T-cells only. This theory may explain why only the decrease in CD-8 T-cell levels was an independent predictor of mortality, although both CD-4 and CD-8 T-cell levels were significantly lower in nonsurvivors in our study. Regardless of the reason, we think that the level of CD-8 T-cells should be monitored at regular intervals in patients with COVID-19 in terms of prognosis. Moreover, treatments that could positively affect CD-8 T-cells should become a current issue, whereas others that could have adverse effects on CD-8 T-cells should be avoided.

The other crucial question is: can cytotoxic T-cell dysfunction be a reason for the ineffective immune response in COVID-19? Obviously, we did not examine any cytokines except for IL-6, which was similar in the CD-8 T-cell groups. Diao et al.^3^ also found that IL-2, IL-4 and INF were similar whereas TNF_µ_, IL-6 and IL-10 were significantly increased in patients with COVID-19. When considering the effect of these cytokines on T-lymphocytes,^4^ these results actually show that T-lymphocytes have enough function. Moreover, it can be thought that excessive TNF_µ_ and IL-10 may be the reason for T-cell exhaustion.

Naturally, the other theory that is more intriguing is CD-8 T-cell exhaustion. Wherry and Kurachi^11^ defined three reasons for CD-8 T-cell exhaustion: persistent antigen exposure, activation of inhibitory receptors, and soluble mediators. The importance of exhaustion in CD-8 T-cells is that it results in the death of these cells, which may explain the decrease in their levels. Persistent antigen exposure and activation of inhibitory receptors on the surface of CD-8 T-cells can be accepted as two logical theories for this exhaustion. It is claimed that being in the chronic phase of viral infection is the main reason for the exhaustion in these two theories.^11,12,13,14^ Thus, it is argued that if effective antiviral agents can be used in the early phase, they can prevent viral infection and CD-8 T-cell exhaustion.^11^ Nevertheless, COVID-19 patients are still exposed to viral loads because there are no effective antiviral agents. The third reason is complicated; however, in our opinion, it is the most important event that should be discussed in patients with COVID-19. Implied soluble mediators are defined as immunosuppressive (IL-10 and TGFß) and inflammatory (IFNs and IL-6) cytokines.^11^ It is clearly known that IL-10 causes exhaustion in CD-8 T-cells and that blockade of IL-10 prevents exhaustion.^15,16^ On the other hand, IFNs are accused of exhaustion, especially in the chronic phase, whereas it is claimed that IL-6 may be required to control chronic viral infection and CD-8 T-cell exhaustion.^11^ In contrast, Diao et al.^3^ concluded that increased IL-6 reduced CD-8 T-cell levels based on a negative correlation between IL-6 levels and CD-8 T-cell levels. However, this negative correlation was quite weak.

Currently, IL-6 blockers are used to prevent the existence of cytokine release syndrome (CRS), which has been made responsible for the worst clinical status in patients with COVID-19.^17,18^ However, the IL-6 level was a poor prognostic factor, and there was no threshold level for it.^19^ In addition, it was demonstrated that there was no relationship between the usage of IL blockers and outcomes, and an increase in the rate of secondary infections could be observed after its administration.^19,20^ In our study, IL-6 levels were similar in all groups. Moreover, in neither all patients nor patients with CD-8 T-cell levels ≤115, there was any effect of the usage of IL-6 blockers on survival. On the other hand, it can be seen that the percentage of the detected microorganism types was similar between the groups. We believe that more immunosuppressed patients could not handle secondary infections, except for COVID-19 infection. Additionally, we demonstrated that the relative risk of having more than one group of microorganisms throughout the ICU stay was greater in patients with CD-8 T-cell levels ≤115 and IL blockers (+).

Now we should ask ourselves this critical question: what is the real reason for the worst clinical status in COVID-19: increased IL-6 levels and CRS or decreased CD-8 T-cell levels and immunosuppression? If there are no effective antiviral agents and detected threshold values for IL-6 to define CRS or if there is a possibility of increased secondary infections caused by the usage of IL-6 blockers, we may contribute to the worst clinical status by ignoring all of them. For these reasons, we strongly believe that CD-8 T-cell levels should be checked especially before IL-6 blocker administration and routinely monitored in patients with COVID-19 to detect prognosis and regulation of treatments.

The most important limitation of this study is the small sample size. Conducting larger studies could make it possible to find thresholds for therapeutic and preventive approaches for this patient group and to be part of the ICU guideline. The lowest CD-8 T-cell level in the first week was included in the study. Repeated measurements may provide new data, especially in patients who require long stays in the ICU.

Age, APACHE-II, and CD-8 T-cell level are independent risk factors for mortality in patients with COVID-19 pneumonia in the ICU. These results bring along the necessity of monitoring CD-8 T-cells and checking their levels before IL blockers are administered. Additionally, in our opinion, monitoring CD-8 T-cells in patients with COVID-19 will ensure that secondary infections and treatments are monitored.

## Figures and Tables

**Table 1:**
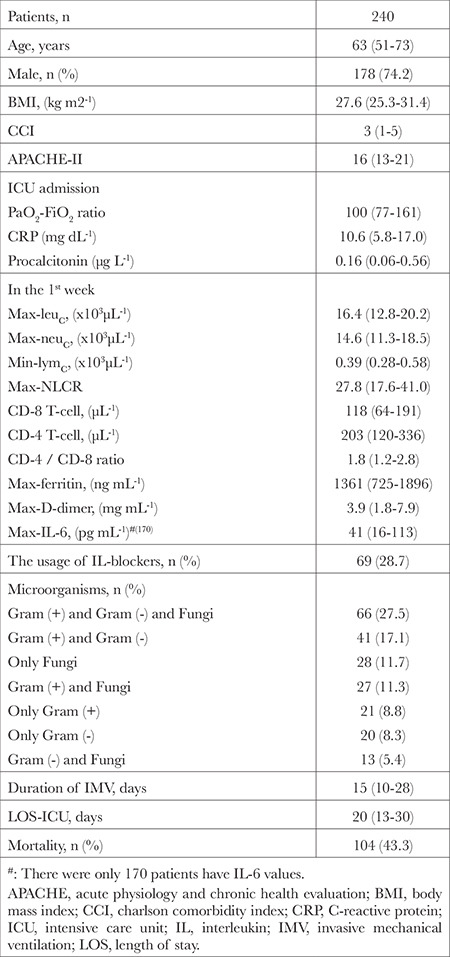
Demographic Data, Laboratories, Therapies and Outcomes

**Table 2:**
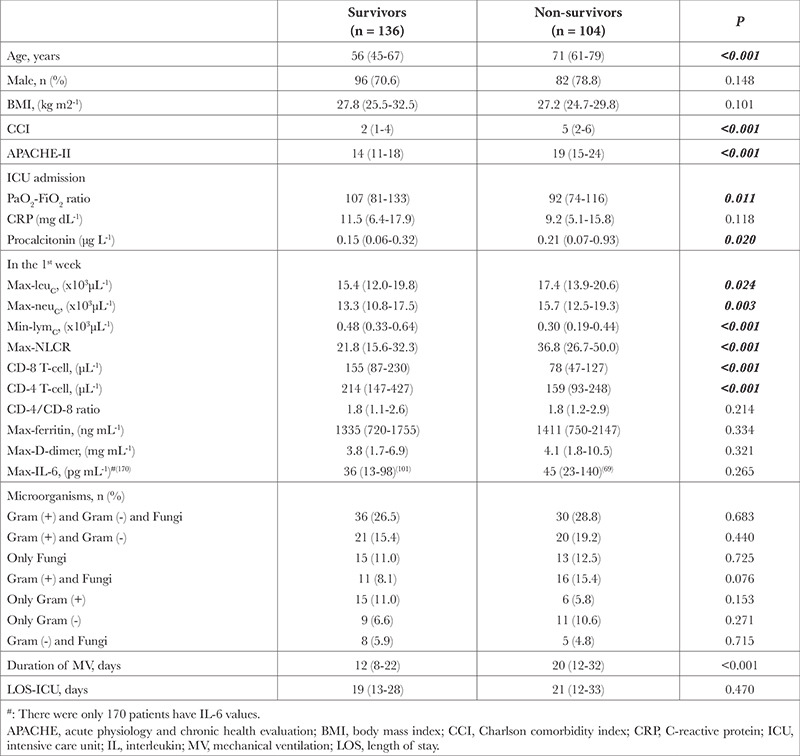
Comparison Between Survivors and Non-survivors

**Table 3:**
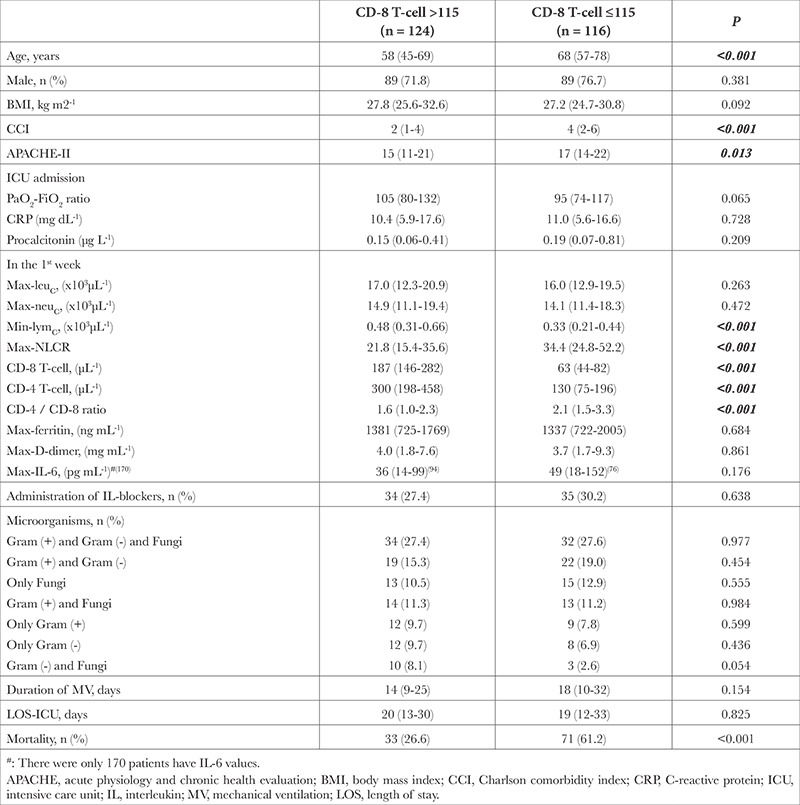
Comparison Between CD-8 T-cell Groups

**Table 4:**
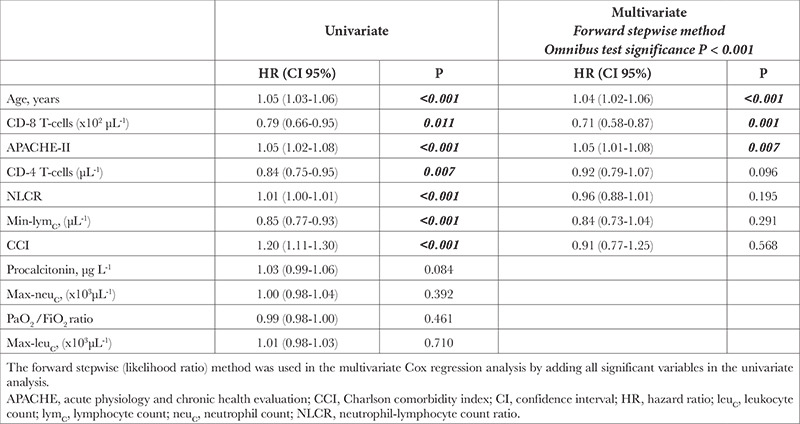
Cox Regression Analyses for the Risk of Mortality
